# The emergence of 3D-printed firearms: An analysis of media and law enforcement reports

**DOI:** 10.1016/j.fsisyn.2024.100464

**Published:** 2024-03-28

**Authors:** Stefan Schaufelbühl, Nicolas Florquin, Denis Werner, Olivier Delémont

**Affiliations:** a Ecole des Sciences criminelles, University of Lausanne, Switzerland; b Small Arms Survey, Graduate Institute of International and Development Studies, Geneva, Switzerland; c Department of Chemistry, Biochemistry and Physics, Université du Québec à Trois-Rivières, Canada; d Groupe de Recherche en Science Forensique (GRSF), Trois-Rivières, Québec, Canada

**Keywords:** Additive manufacturing, Craft-produced firearms, Fully 3D-printed (F3DP) firearms, Hybrid firearms, Parts kit completions/conversions (PKC)

## Abstract

3D-printed firearms, an emerging category of privately made firearms (PMF) produced beyond government control, have become increasingly prevalent due to technological advancements. They are now emerging as a cost-effective and reliable alternative to conventional firearms. Raised to public awareness following the 2013 release of the 3D-printed Liberator, these firearms are now more commonly encountered by police forces.

This article analyses various reports involving 3D-printed firearms, reflecting the increasing encounters by law enforcement agencies. It examines 186 cases involving 3D-printed firearms, primarily from North America, Europe, and Oceania, highlighting a significant rise in incidents since 2021. These incidents include seizures, illicit uses, and online sales, with the firearms typically being hybrid models, Parts Kit Completions/Conversions (PKC), or firearm components such as auto sears. The study underscores the use of affordable equipment and materials for production, emphasizing the accessibility and potential risks of these firearms.

## Highlights

• Data on 186 cases involving 3D-printed firearms and parts was collected.
• 92 % of the reported cases took place in either North America, Europe or Oceania.
• A sharp increase in reported cases was observed since the year 2021.
• Hybrid firearms, PKC builds, and auto sears are the most seized 3D-printed objects.
• Producers mostly use entry-level equipment, and no high-end material is necessary.

## 1 Introduction

Improvised and homemade firearms are available in various parts of the world. While they generally remain outnumbered by industrially produced firearms, trends in recent years point to the growing weight of improvised armaments in both conflict and crime settings. Ingenious blacksmiths in diverse regions including Pakistan, the Philippines, China and West Africa have produced homemade craft firearms for decades, the sophistication of which has increased over time [1,2]. In Europe, blank-firing alarm handguns illegally converted to shoot lethal projectiles represent one of the main sources of illicit firearms [3]. In the United States, so-called “privately made firearms” (PMF) account for a growing proportion of firearms used in crime: from 2016 to 2021, the “*ATF received approximately 45,240 reports of suspected privately made firearms recovered by law enforcement, including in 692 homicide or attempted homicide investigations*” [4]. Around the world, PMF encompass a variety of types, ranging from rudimentary and improvised designs to more sophisticated or craft-produced firearms, including among others zip guns, converted, modified, 3D-printed firearms, as well as 80 % frames and lowers. The origins of these firearms can be traced back to the world wars, with their prominence growing towards the end of the last century and persisting to the present [1]. Especially in recent years, the advent of 3D-printed firearms has emerged as a new phenomenon, gaining significant attention through all sorts of online media [5,6].

These various types of PMF have in common that their production can be performed in the physical, geographical, and temporal proximity of the end user, whether for criminal purposes or not. Efforts to control international firearms transfers and prevent illicit cross-border trafficking of firearms are therefore rendered largely irrelevant as criminals are able to either produce or acquire these firearms locally and outside of any state control. Despite the opportunity to produce locally, there is the potential for trade to be conducted across state and national borders. Traditional methods to investigate and trace these homemade firearms are challenged by the absence of serial numbers. Furthermore, 3D-printed firearms often present less discernible, or sometimes even absent, ballistic traces, further complicating their investigation [3,7]. Among the wide range of PMF, 3D-printed firearms bring unique challenges to firearms regulations. Indeed, the last generation models are made primarily or entirely from 3D-printed components and other widely available commercial materials, further complicating control and tracing efforts [8]. Additive manufacturing (i.e., 3D printing technology and materials) is increasingly affordable and available, and the models of 3D-printed firearms produced are more and more adaptable and reliable as well as capable, for instance, of fully automatic fire. In spite of this growing threat, there are no systematic efforts to date to document and map the proliferation of 3D-printed firearms worldwide, including cases of production, seizures, and illicit use.

This article is an initial attempt to fill this gap by reviewing and mapping out cases reported in the media and other publicly available sources. The examination of various sources and reports was conducted jointly and comprehensively, providing an understanding of the circumstances and contexts in which 3D-printed firearms were found. Other aspects, such as the types of seized firearms or their production, are also examined. The aim of this article is to create a picture of the current situation of 3D-printed firearms that will contribute to providing relevant insights into this subject area, including possible trends in production, trafficking and use.

## 2 Methodology

### 2.1 Data gathering

A relevant case is an event in which illicit activities – that is, activities that violate applicable national or international law – were carried out involving 3D-printed firearms or parts. This encompasses cases of various kinds corresponding in particular to seizure, use, sale offer and buyback of such 3D-printed items. Even though buybacks are state programmes and therefore not illicit activities, it has been decided to include these in the cases as they reflect the presence of 3D printed firearms and components as well as illustrate prevention methods applied by the police.

The research was conducted exclusively on the Clear Web and focused on publicly available information. Using keywords in different languages, mainly English, German, Spanish and French, reports mentioning 3D-printed firearms or parts were sought using common search engines such as Google, Bing, or DuckDuckGo. In a few cases, searches were also carried out in the language of the region or country in which the case was originally reported, sometimes allowing to complement the available data. Furthermore, searches were also made on various blogs (e.g., 3Dprint.com, The Firearm Blog) and Reddit. Since many law enforcement agencies have their own pages on social media (e.g., X formerly known as Twitter, Facebook or Instagram), reports were also searched for on these platforms. This study also incorporated data from cases obtained through personal communications with forensic and firearms experts. While this information is not publicly available, its inclusion was considered important to corroborate statements and enrich the study's overall value. These cases have only a minor impact on the observed trends, as they represent only around 5 % of the total cases analysed.

Once a relevant case was found, it was assigned a unique code for identification. A PDF of the information was created and all other resources, such as images and videos, were downloaded and stored locally. The data collection itself took place over the period from February 2021 to August 2023. In order to avoid including several reports of the same incidents in the data analysis, a preliminary pre-processing of the data was carried out to exclude duplicates of the same case.

### 2.2 Data analysis

The collected data was organised in a Microsoft Excel (version 2301) document and analysed for different criteria. Spatial (city, region, country, continent) and temporal data were recorded to allow the analysis of the history and development of such cases worldwide. More details were also sought on the type of item(s) involved in each case. For this purpose, the items were divided into three main categories: 3D-printed firearms, imitation firearms and firearm parts. The 3D-printed firearm category was further subdivided according to criteria defined in another published report by Armament Research Services (ARES) to more precisely describe the type of 3D-printed firearm^1^: Fully 3D-printed (F3DP) firearms, hybrid firearms and Parts Kit Completions/Conversions (PKC) [9]. If possible, the exact model of the item was determined. The circumstances of each case were described in more detail in a free text section, in order to determine the nature of activities that these 3D-printed items were involved in. Lastly, details on the manufacturing equipment used to fabricate the items were studied. Thus, the information about 3D printing material such as equipment encompasses the 3D printers and the printing material (filament and resin).

Data processing and data visualisation were undertaken using the functions available in Microsoft Excel. Tableau Desktop (version 2022.4.0) was used to visualise the spatial data. For the normalization of the data per 100,000 inhabitants, population figures from August 2021 were used [10].

## 3 Results

In total, data on 186 cases was gathered by searching newspaper articles, police reports, posts on social media, and through personal communication. All cases occurred in a period between 2014 and August 2023. In the following sections, individual aspects of the research are presented in more detail. Due to the different sources and details available on the cases, it was not always possible to extract the exact same information from all cases. In any case, efforts have consistently been made to extract a maximum of open-source information from the available sources (text, images or videos) in an attempt to standardise the data for analysis.

Before delving into the results, it is important to address certain aspects regarding their interpretation. The results and figures, drawn from press reports and other relevant sources, may not accurately reflect the actual reality due to the non-publication of many cases. Influenced by socio-political factors, such as the freedom of press and police publication policies, as well as the languages used in the data search, these results require critical contextualisation by the reader. The data highlight the fact that 3D-printed firearms are an emerging problem for law enforcement agencies in various geographical and cultural settings, although the true extent and relative distribution of the problem is not necessarily shown.

### 3.1 Temporal trend

During the research, many different dates were recorded referring either to the arrest of a suspect, seizure of material, publication of the article (newspaper, social media or announcement by the law enforcement agencies) or the conviction of a person. Where possible, the date of arrest/seizure was prioritised as this best reflected when the cases took place. This was not always possible and thus the other available data was sometimes used. For some cases registered through personal communication, only the year of the case could be assessed. On account of these points, it was decided that the case numbers would be broken down by year and not in smaller time intervals (e.g., half-year or quarters). This makes it possible to compensate for the differences that sometimes exist between the data of individual cases.

**Fig. 1:**
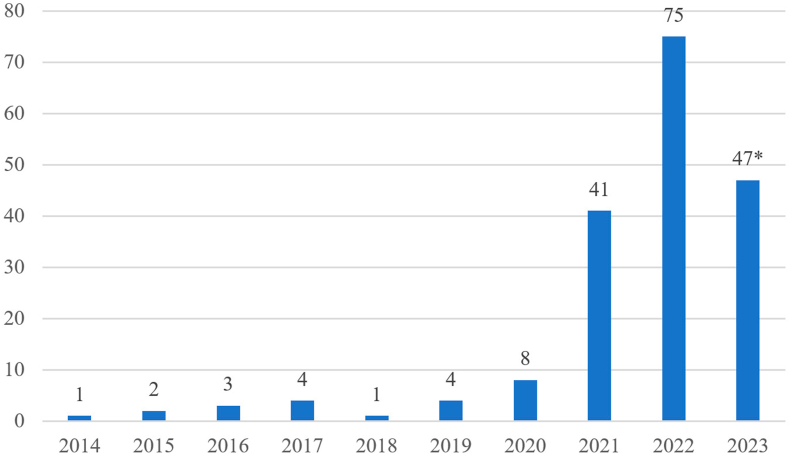
Development of global case numbers per year based on open-source information from available media and law enforcement reports. * The numbers from 2023 cover the period up to August.

The evolution of the cases is visualised in Fig. 1. The first case was reported from Japan in mid-2014, in which a man was arrested for possession of several 3D-printed firearms, presumably of the type of F3DP firearms [11]. Over the next few years until 2020, the number of cases remained in the single digits. From 2021 onwards, however, the number of cases increased sharply and was in the mid to high double-digit range. The last case detected and included in our dataset occurred on August 22^nd^, 2023. This increase cannot be attributed to a single event, but several points can partly explain it. First, cases were initially reported only sporadically. Since 2021, the number of published police reports on such cases increased, resulting in an upswing in media attention on this topic. Secondly, the steadily growing community of 3D-printed firearm enthusiasts gained more and more ground on the Internet. The exchange of expertise and experience as well as digital plans on the Internet became increasingly easier. Although platforms like YouTube and Reddit restricted this exchange through new policy changes, the community found other sites that continued to enable this exchange [9]. As a result, file-sharing hosts like LBRY or Odysee or communication platforms like Keybase or Rocket.Chat became new rallying points where the exchange could continue. Lastly, another possible reason is the emergence of more reliable 3D-printed firearms. With the advent of PKC or hybrid firearms around the turn of the decade, the performance of such privately-made firearms could be improved [9,12]. Progress over the years has gone from exploding single-shot firearms to today's semi-automatic or even fully automatic firearms. In addition, the relative ease of manufacturing such firearms and the improvement of their manufacturing instructions and documentation also contributed to the growing interest of more and more people.

### 3.2 Spatial distribution

**Fig. 2:**
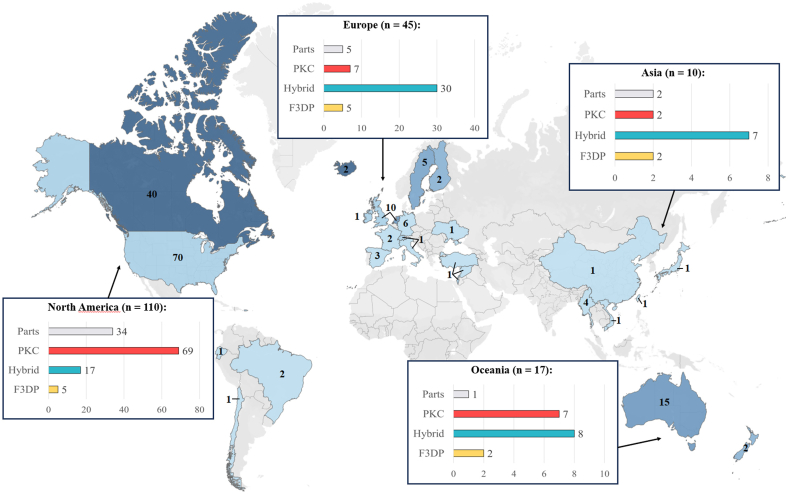
Cases involving 3D-printed firearms or firearm parts per 100,000 population. As the two cases reported in Iceland would have greatly distorted the global picture by normalising to a population of 100,000, the colour gradient was limited to the second-highest value – Canada – allowing a better comparison between the remaining countries. The numbers inside a country represent the absolute number of cases. The information boxes provide details about the total number of cases between brackets of the region and the type and number of items encountered.

Cases of 3D-printed firearms or firearm parts have been reported on almost all continents. There were no reports found on the African continent. The fewest cases were attributed to South America (n = 4) and Asia (n = 10). More cases were registered in Oceania (n = 17), Europe (n = 45) and North America (n = 110). This adds up to 92 % of all cases that can be attributed to those three continents. The distribution of cases, normalised to 100,000 population (colour gradient) and the absolute number of cases per country (labels inside countries), among the different countries is visualised in Fig. 2.^2^ Given the two cases assigned to Iceland, the colour gradient on the graphic map has been adjusted to prevent significant distortion of representation. Due to Iceland's low population and the normalization of the data, it emerged as an outlier, which compromised the comparability with and between other countries. It can be seen that the number of cases per 100,000 inhabitants is generally low. In most European countries the rate is less than 0.01, countries like Great Britain, Finland, Iceland, Ireland, Netherlands and Sweden have higher rates than other European countries. In comparison, the number per 100,000 inhabitants was about 0.02 in the USA, 0.10 in Canada, 0.06 in Australia and 0.04 in New Zealand. In absolute numbers, most cases were publicly reported in the USA (n = 70), Canada (n = 40) and Australia (n = 15). The Netherlands and Great Britain are the only countries in Europe with a double-digit number of cases (n = 10). While the number of Great Britain is mainly composed of published reports on seizures by the police, several sale offers were found in the Netherlands. In all other countries, the number of cases was in single digits. It can be observed that such events generally took place in regions of western culture. However, this distribution can be explained by the fact that primarily media and reports from these regions were found on the Internet. In conclusion, although it is apparent from this data that the production or use of 3D-printed firearms appears to be marginal, it nevertheless shows that it is a trend that is now widespread throughout the world.

### 3.3 Type of cases

The cases were assigned to a total of five categories in order to describe the context of each case. These categories are as follows: *Seizure*, *Discharged firearm*, *Buyback*, *Sale offer* and *Other*.

Cases in which 3D-printed firearms, firearm parts and equipment were recovered by the police and law enforcement services were labelled as *Seizure,* regardless of the circumstances in which they were found. No discharged firearms were reported among them. There were 164 cases assigned to this category which corresponds to 88 % of the dataset. The number and type of objects seized could differ considerably between cases. 3D-printed firearms were confiscated in most cases. In a small two-digit number of cases, it occurred that only 3D-printed firearm parts were confiscated. Reports ranged from only a single 3D-printed object seized to a large number of 3D-printed firearms, including manufacturing equipment. Due to the diverse quality and quantity of information, it proved challenging to determine the exact number of 3D-printed firearms. The following figures are therefore only approximate. In about a third of these cases, it was mentioned that only a single 3D-printed firearm was involved. In another third, at least two 3D-printed firearms were involved, but the cases in which more than 10 3D-printed firearms were discovered numbered in the single digits. In almost 30 cases, it was not possible to determine the number of 3D-printed firearms involved due to the lack of information and data. Cases involving a larger number of 3D-printed firearms or parts could sometimes be assigned to a context of gang delinquency or possible sales activities. A notable aspect is the discoveries of workshops where the production of 3D-printed products can take place on a larger scale. In such environments, the potential to reveal information about the manufacture of the 3D-printed firearms is at its highest. Compared to cases where only a 3D-printed firearm is found, information on manufacturing remains elusive to investigators for the time being.

Cases assigned to the category *Discharged firearm* include instances in which 3D-printed firearms were discharged. These encompass any circumstances: unintentional or deliberate discharges of 3D-printed firearms with or without harm to persons. A total of eight reports of such incidents were encountered. Six of them came from the USA and one each from Canada and Iceland. In two cases, parts were used to convert semi-automatic (conventional) firearms into fully automatic firearms using 3D-printed auto sears, in two others printed pistol frames were used and in one case an FGC-9 (hybrid firearm). In the other cases, there were indications of firearms made using 3D printers, but further details were not available.

*Buyback* is the term given to events where people can turn in 3D-printed firearms to the state with impunity and occasional compensation. Three entries were recorded, all of which took place within the USA. Among the returned items were 3D-printed firearms (F3DP, hybrid and PKC)^3^ and parts (auto sears). One instance highlighted how means to control firearms can be exploited by returning fast-produced 3D-printed objects in exchange for compensation. An X (formerly known as Twitter) user pointed out that it earned him 21,000 USD in gift cards by turning in 3D-printed firearms and parts and mocked the authorities’ actions [13]. According to the same source, the material costs amounted to around 50 USD (the price of the 3D printer is not included). As a result of this, the rules of such buybacks have been changed. This shows all the more how complicated the handling of 3D-printed firearms can be for authorities.

*Sale offer* refers to the sale of 3D-printed firearms or parts. A total of four cases were assigned to this category, all of which took place within Europe. Unless the sellers had authorisation for the production and sale of 3D-printed firearms, this activity occurred within an illegal framework. This could not be verified. Telegram was among the networks used for the sales, which is a known platform on which illegal activities are carried out with relatively little intervention from its developers. In all four cases, FGC-9 were offered for sale. The prices ranged from 900 to 2500 EUR. One source also offered a modified Songbird single-shot pistol alongside an FGC-9, but no price was given.

The category *Other* regroups seven cases that could not be unambiguously assigned to another category. Four of them could be assigned to the use of 3D printing technology in conflict zones. The 3D-printed items included 3D-printed firearms or attachments for other firearms. The use of FGC-9 by anti-government forces in the ongoing civil war in Myanmar was reported [14]. A video from the same conflict surfaced on Reddit showing the alleged use of a FGC-9 in combat [15]. 3D printers were also used in Ukraine, where fins for grenades dropped from drones are produced, or in Syria, where parts for firearms and other weapons were produced [16,17]. Another incident was about the sighting of an FGC-9 during an announcement by an Irish paramilitary group. No further reports have been found on this incident so far [18]. Two more cases from Europe were also registered within this category. A Swiss journalist had a Liberator illegally manufactured for a story, while in England a man was convicted for possession of files of 3D-printed firearms [19,20].

### 3.4 Encountered types of items

The seized objects were divided into three different main categories: *3D-printed firearms*, *Imitation firearms* and *Firearm parts*. A *3D-printed firearm* is defined here as an object that is intended to be capable of discharging ammunition and that was made using 3D printing technology. The actual functionality as well as the state after any discharge were not considered. This category was further divided into three sub-categories according to the criteria presented in an Armament Research Services (ARES) report: *Fully 3D-printed* (*F3DP*) *firearms*, *Hybrid firearms* and *Parts* *kit* *completions/conversions* (*PKC*) [9]. Making the distinction between F3DP and hybrid firearms may vary depending on the source and experts as these categories may overlap slightly. In this work, 3D-printed firearms classified as hybrids are those that have integrated non-printed parts that impact positively on the performance and reliability of the 3D-printed firearms. Examples are metal tubes used as barrels or compression springs that power the firing pin. An imitation firearm is counted as a replica that resembles a real firearm, but which is not designed for the discharge of ammunition. The final object – the imitation firearm – is often printed at once and thus not functional. The category of firearm parts includes 3D-printed parts that have been manufactured with 3D-printing technology and that can be attached to a (conventional) firearm or other arms and weapon platforms (e.g., grenades, drones).

**Fig. 3:**
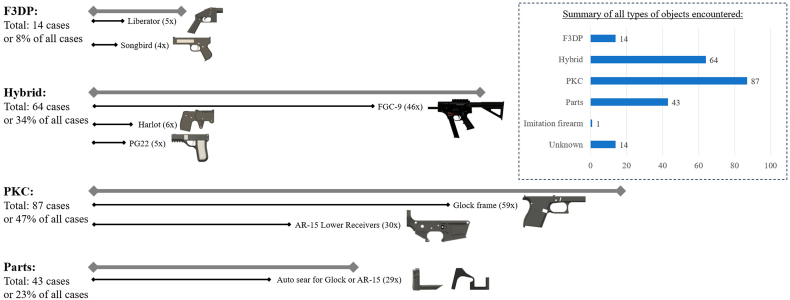
Number of occurrences for the 3D-printed firearms and firearm parts categories in the reports. The left side of the graph illustrates the most commonly encountered firearm models in the F3DP, Hybrid and PKC categories as well as firearm parts. Note that the number of items (i.e., firearms and parts) encountered may be higher than the total number of cases, as several different items may be found per case. For example, the FGC-9 alone has been encountered in 46 cases out of the total of 64 cases in which hybrid firearms were reported.

Due to a lack of information, it was not possible to determine the exact number of objects involved in each case. Either the exact number could not be taken from the source text, or the available image and video material was not sufficient to determine it on our own. For these reasons, it was decided that only the occurrence of the different categories per case would be determined. This means that in a case where three identical firearms were recovered, they were only counted as one occurrence. An overview of what was found is shown in Fig. 3. It was possible that different types of objects were observed or reported for a single case. Therefore, the sum of all occurrences exceeds the total number of cases.

The category of F3DP firearms has the fewest occurrences, as only 14 cases were recorded for these. Models such as the well-known Liberator (.380 ACP) or the PM422 Songbird (.22 LR) appeared in several cases each. However, other models such as the PM522 Washbear (.22 LR) as well as the Grizzly (.22 LR) were also seen. These 3D-printed firearms are mainly made of 3D-printed parts, but resort to some non-printed parts (e.g., a nail as firing pin or elastic bands to power components). They are considered less reliable and durable, which has also been shown in various studies [9,[21], [22], [23]]. Hybrid firearms were much more frequently observed with 64 occurrences in cases. Unlike F3DP firearms, these are additionally equipped with non-printed and non-regulated components (e.g., metal tubes as barrels or other reinforcements), which are designed to withstand the pressure of a discharge more efficiently and thus be more reliable and durable. The FGC-9 designed by JStark1809 appeared most frequently by a wide margin (n = 46). FGC stands for “Fuck Gun Control” and the number 9 refers to the 9 × 19 mm Parabellum calibre. This is considered a revolutionary design and can almost be compared to conventional firearms because of the improved performance and durability [5,24]. Meanwhile, several variants of the FGC-9 which are either improved versions of the base model (e.g., MKI and MKII models) or with other specifications (e.g., calibre of .22 LR) have been released on the Internet. Other designs, like the Harlot (.22 LR) or the PG22 (.22 Short) single-shot pistols, were also observed in some cases. These are handguns that differ from F3DP firearms by being fitted with springs, screws and .22 calibre rifled barrel liners or steel tubes as barrels. PKC are firearms in which the majority of the components are sourced from conventional firearms (e.g., fire control group, barrel, etc.) but assembled using 3D-printed receivers or frames. The performance in terms of ballistic and reliability of said 3D-printed firearms can reach the same level as the one of conventional firearms [9]. This category mainly comprises designs of Glock pistol frames and AR-15 lower receivers. The selection is not limited to just one design, but a variety of designs and models are freely available on the Internet. Pistol frames of a S&W model SW40 (.40 S&W) and a FN model FNS-40 (.40 S&W) were also found. However, in approximately five cases the type of pistol could not be precisely determined. Compared to F3DP or hybrid firearms, PKC builds are the most common type of items found in all these cases (n = 87).

In the category of firearm parts, 3D-printed auto sears, sometimes also referred to as giggle switches, were mainly represented. These components are add-on parts that can convert semi-automatic (conventional) firearms into fully automatic firearms. 3D-printed auto sears were mainly observed for Glock pistols and AR-15 rifles. The production as well as the installation of such components require relatively little time and effort, but considerably increase the firepower of a firearm. Other 3D-printed attachments that were often found in combination with other conventional firearms included stocks, grips and silencers.

Of the total of 186 cases, in 14 of them the type of objects seized could not be determined more precisely. These could be either 3D-printed firearms or parts which, however, remained undisclosed. In only one case was it reported that imitation firearms, including copies of Glock, SIG and Colt 1911 pistols, were found by the police.

### 3.5 Equipment used in the fabrication

In 62 cases, information on the equipment used – i.e., printers and printing material – was collected. For the printers, based on the available information, efforts were made to determine the printing process, manufacturer and model. In some cases, it was not possible to determine all or even any of these points, as no picture material was available, and it could only be inferred from the text that one or more printers had been confiscated. The total number of printers is 106, with 18 cases mentioning more than one printer involved. For example, nine printers of the same model were found during the recovery of a workshop in the Netherlands in 2021 [25]. In Finland, a workshop with at least five of the same printers was also discovered in the same year [26]. The determined 3D printer models as well as their number of occurrences are summarised in Table 1. When classifying the printing processes defined by the ISO/ASTM 52900 standard, material extrusion (MEX) systems were used the most (n = 93). A small number (n = 3) is vat photopolymerisation (VPP) printers, which utilise resin as the printing material. These results reflect what is reported in the Wohlers Report: MEX printers dominate the 3D printing systems used in the private sector [27]. Of the 106 printers, a total of 14 different manufacturers and 17 different models could be determined. For 21 printers, it was not possible to determine the manufacturer and the model. The manufacturer Creality (n = 58) appeared most prominently. Their Ender 3 models (n = 52), which are often praised for their affordability, ease of use and modifiability, dominate the list [28]. Other brands of printers were also found, but their frequency was much lower than that of the Ender 3. Other well-known brands could also be determined, such as Prusa and its well-known i3 MK3 model series, as well as Anycubic which received a lot of publicity for their affordable VPP printers. All these models are desktop printers, which according to the definition in the Wohlers Report cost less than 5000 USD and are primarily intended for the private sector [27].

**Table 1:** Manufacturer and models of 3D printers determined with the available resources.

Manufacturer	Printing process	Model	Occurrences
Anycubic (China)	MEX	Mega series	3
	VPP	Photon series	2
Bambulab (China)	MEX	X1	2
BIQU (China)	MEX	B1	3
BQ (Spain)	MEX	Witbox 2	1
Bresser (Germany)	MEX	Rex	1
Creality (China)	MEX	Ender 3 series	52
		Ender 5 series	5
		CR-10	1
Elegoo (China)	VPP	Mars 2P	1
Flashforge (China)	MEX	Adventurer 4	1
Prusa (Czech Republic)	MEX	i3 series	3
Qidi (China)	MEX	X-Plus II	1
Ultimaker (Netherlands)	MEX	S3 or S5	1
Voxelab (China)	MEX	Aquila	1
Wizmaker (China)	MEX	P1	1
XYZ Printing (USA)	MEX	Da Vinci	6
Unknown	MEX	Unknown	11
Unknown	Unknown	Unknown	10

Information was also sought regarding the printing material. In these cases, these were in the form of filament for MEX printers or in the form of resin for VPP printers. Unfortunately, the results were much sparser. In only 13 cases could precise information on the material be obtained. In some other cases, printing material could also be observed on the image material, but due to insufficient quality, the manufacturer and type could not be determined. A total of five manufacturers could be identified: Duramic (USA), eSun (China), Overture (USA), Polymaker (China) and Protech (Sweden). The printing material is in the form of filament. Only PLA type material and modified PLA (PLA+ from eSun and Duramic, PLA Pro from Overture) were observed. Printing material used for VPP could not be determined and no information on this can be given.

In a small number of cases, images of the scene of investigation were released showing the environment in which the production was carried out. Everything could be found from seemingly disorganised to structured and orderly environments. It is important to note that in such environments, in addition to the printers and printing material, a large number of items required for the manufacturing process can be discovered as well. The manufacturing process requires different materials for different stages. Electronic devices (e.g., computer, tablet, smartphone) are involved for acquiring the digital files of the 3D-printed items and for the use of the dedicated software. Different tools (e.g., hex keys, screwdrivers) and consumables (e.g., alcohol for cleaning, tape, hairspray or glue for improved printing quality) can be used for the preparation of the 3D printer. Especially after printing and during the post-processing phase of the parts, a variety of tools, machines and consumables come into play. Not to be forgotten are those parts that are not printed but can be incorporated into the 3D-printed firearm (e.g., metal tubes, springs, magazines, etc.). These elements potentially contain traces of different types (digital or physical) which cannot be neglected for an investigation. Ultimately and despite the variety and considerable quantity of materials required in the manufacturing process, all of this can be accommodated in a relatively compact space, which further complicates the detection of such activity by law enforcement.

## 4 Discussion

The majority of the data was collected through research on the Internet (Clear Web). Official reports by government institutions (e.g., police or public prosecution) were prioritised as these are closest to the real facts and thus present the most trustworthy information. Such reports were often found on the official page of the state organ or on their social media account (e.g., X formerly known as Twitter or Facebook). Cases communicated through in-person communication were assigned to the same level of trust. The proportion of cases reported through these two means was 43 %. The information acquired through other means did not come from official sources, which required them to be looked at somewhat more critically. Attempts were made to find several reports of the same case and cross-reference the information they contained. By doing so, confidence in the information was increased. Especially picture and video material were of great importance in obtaining information, through which further details (e.g., the type of object or equipment used to produce it) were manually coded. An example that shows that wariness is required with such reports is the attack in Halle, Germany in 2019. Various sources initially reported that the perpetrator used firearms, some of which were assembled with the help of 3D printing, in the attack itself. However, this turned out to be untrue and the concerned articles have been modified since. The proportion of cases that could be found in publicly available newspapers was 46 %. Information from blog entries and social networks also had to be considered more critically and accounted for 11 %.

This project largely relied on the availability of information in the public media. It is likely that only a fraction of cases is publicly disclosed, due to differences in criminal policy strategies and communication policies between police forces, or for other socio-political reasons in the regions concerned. Official databases on a national or international level are also not maintained or accessible. The language constraints in data searches also impacted the volume of information obtained. As a consequence, the overview presented here is based primarily on publicly available information and does not reflect the complete picture. Despite this limitation, an interesting overview of the current situation has been drawn from the 186 cases collected mostly in open source. The results presented contribute to the understanding of several points related to spatial-temporal trends, 3D-printed firearms and parts, as well as the production of these objects.

**Fig. 4:**
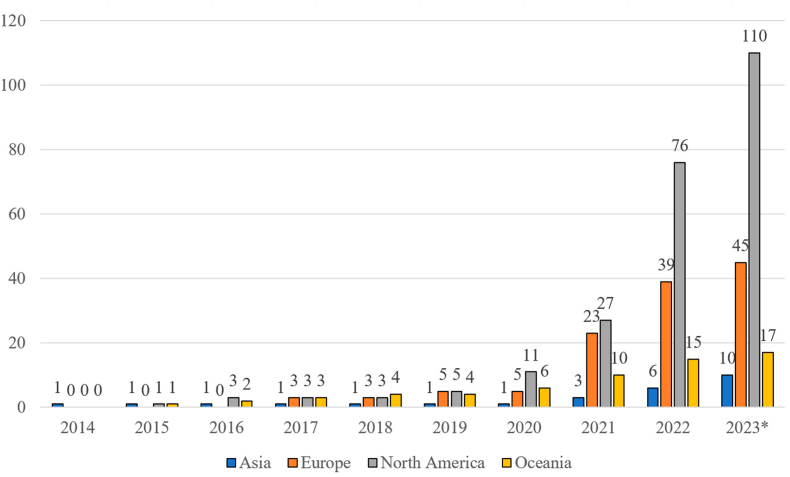
*Sum of number of cases for four regions: Asia, Europe, North America, and Oceania*. * Note that the collection of cases was completed in August 2023 and no further cases were recorded after this date. This explains the stagnation of cases for the year 2023.

With regard to spatial-temporal information, further observations have been made. The number of cases and their evolution over time can vary from one region to another. For example, different rates of increase were observed for the three regions of Oceania, Europe and North America. It can be noticed that the number of cases grows only slightly from a rather low level until 2020 and then from 2021 onwards, the growth is more or less significant depending on the region (see Fig. 4). In North America, the number more than doubled in 2021 and 2022. In Europe, however, the number of cases jumped in 2021 and grew at the same rate in 2022 as it did the previous year. In Oceania, the increase was also constant, but with a lower rate than in the other two regions.

It also seemed that public reporting by state organs is much more prevalent in North America than in Europe. Almost two thirds of all European cases were collected through unofficial sources (online newspapers, blogs, social media). Around a third was gathered through official channels (police or personal communication with experts). In North America, on the other hand, the proportion of cases registered through official sources is at 50 %. It was observed that such announcements are communicated more often in said region than is the case in other regions looked at in this work. In the USA, access to such cases is also facilitated by PACER (Public Access to Court Electronic Records), which can have a positive effect on the number of cases. This highlights differences in reporting between regions and may partly explain why differences exist between regions.

In terms of the type of cases, reports about seizures of 3D-printed firearms and sometimes manufacturing equipment are dominant. Little has been reported about the actual use with or without harmed persons, or even about the trafficking of 3D-printed firearms. There may be two explanations for this. Firstly, this can be justified by authorities’ strategy to publicly report such cases. Publication may be waived if sensitive data is involved or non-balanced reporting by media is to be prevented. Secondly, it could be that 3D-printed firearms have in fact only been used very rarely. In the end, little can be said about the actual use of this type of firearm at the moment. As already mentioned, seizures can include only a single item or a variety of materials (firearms, parts and manufacturing equipment). As a result, in some cases nothing is known about the manufacture at the outset, while in others the equipment potentially used for the manufacture is available along with it. However, the question of the origin of a seized 3D-printed object should always be investigated. It is not surprising, however, that such 3D-printed firearms are also offered for sale on the Internet. Even though the number of file-sharing sites has increased at a progressive rate, an already published study showed that the trade related to such 3D-printed firearms also takes place on the dark web [29]. Depending on the region, a price of up to 2500 EUR can be charged for an FGC-9. This price for a PMF seems high but is still below the average price of a conventional sub machine gun that can be bought on the darknet [30,31]. Offers for parts were not found. However, it can be assumed that these are also traded. Especially for 3D-printed auto sears, which can be produced easily and quickly, the price can probably be kept relatively low. Finally, the use of 3D printers in zones of conflict has been confirmed. Such technology can be used for repairs, innovations, improvised devices or in case of supply problems while also allowing a certain degree of flexibility. Deployments of 3D printing technology are known from war zones such as Ukraine or Myanmar. A blog entry published in spring 2023 impressively showed how a workshop in a Syrian province has efficiently produced components for various weapons systems in recent years [17]. Although the full extent is difficult to determine, it can be assumed that this type of manufacturing will be gaining momentum in such contexts.

**Fig. 5:**
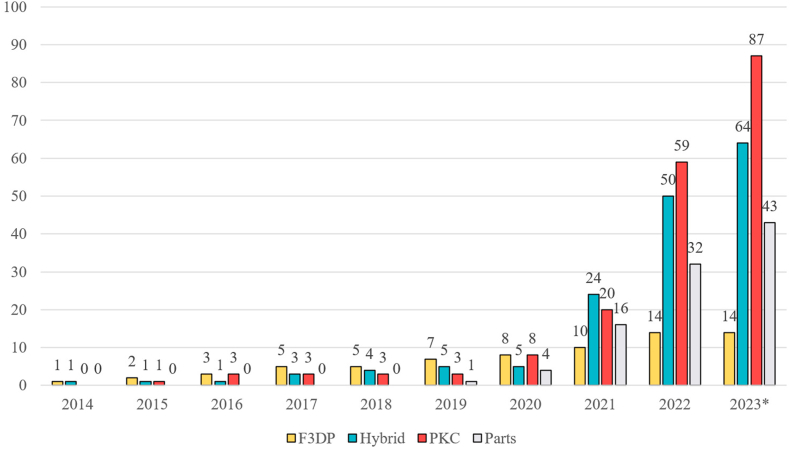
Development of the number of cases in relation to the reported type of 3D-printed object. Unknown types of 3D-printed objects as well as imitation firearms were excluded from this graph due to their low number. * Note that the collection of cases was completed in August 2023 and no further cases were recorded after this date. This explains the stagnation of cases for the year 2023.

All three categories of 3D-printed firearms (F3DP, hybrid firearms and PKC) and parts could be observed in the available data. The exact model of the 3D-printed firearm could not always be determined. This was due to missing information or 3D-printed firearm models that remained unknown despite searches. Regarding the development of the number of reported objects per case and year, this could vary from one type of object to another (see Fig. 5). The increase in F3DP firearms remained consistently modest over the documented years. It was not until 2021 that a total of 10 cases involving such 3D-printed firearms were discovered for the first time. In contrast, the other three visualised categories also exceeded the 10 marks in 2021, but a much stronger increase was subsequently observed. The fact that these 3D-printed firearms are considered more reliable and perform better certainly affected the observed increase.

Differences between regions were also observed in regard to the occurrence of the different types of 3D-printed firearms and firearm components encountered (see Fig. 2). It is well known that gun laws in the USA are more liberal than their European counterparts. The majority of PKC firearms were observed in the USA. As mentioned earlier, these are 3D-printed firearms that use a printed frame or receiver. According to the laws in the USA, manufacturers and importers are required to mark any frame or receiver with a unique serial number, making it the only regulated firearm component by law [32]. This is therefore the sole component of a firearm that is submitted to control to state authorities. All other components, such as the breech, barrel fire control groups and so on, are not marked by serial numbers and can be legally acquired without control. In this context, by 3D printing the only regulated component of a firearm – which is the frame or receiver – government control can be bypassed, and a high-performance firearm can be assembled. The completed firearm then exists beyond any control of the state. In contrast to the US gun laws, European laws are more stringent. For example, in Swiss and German gun laws, other components such as the breech and barrel are considered essential and are subject to a serial number [33,34]. In the United Kingdom, the Firearms Act 1968 bans the manufacturing, purchase, sale and possession of any component part of 3D-printed firearms without government approval [35]. This makes the assembly of PKC models more difficult and much less common in European countries. Due to such laws, the focus shifts to hybrid firearms, which have indeed accounted for the majority of reported 3D-printed firearms in other regions (Europe and Oceania).

As described in the last sections, differences between the regions are evident in the number of cases reported as well as in the type of objects involved. This can be explained by several factors. A first factor is the applicable law in the respective regions, which regulates access to firearms as well as the production of 3D-printed firearms differently, ranging from very permissive regulations to highly restrictive ones. In the US, known for its generally permissive firearms laws, there has been a shift in several states towards more restrictive regulations regarding 3D-printed firearms [36]. For example, in New South Wales, Australia, restrictive regulations have been introduced, rendering the possession of the digital plans a punishable offense [37]. Conversely, many countries do not deem the possession of these plans illegal but impose restrictions on the production of firearms of any kind (conventional or PMF). However, these broader prohibitions still cover the production and use of 3D-printed firearms. Access to conventional firearms, whether highly restricted or liberal, may also have an impact on the increase in cases involving 3D-printed firearms. If access is severely restricted, there is more likely to be a need to resort to an alternative, such as 3D-printed firearms. This is the case in Australia, for example, where gun laws remain stringent. Another factor, already mentioned above, is the reporting of such cases by police forces. Different cultures and principles influence the extent to which such cases are reported. In countries such as the USA or Canada, increased activity in this kind of reporting has been observed. The factors mentioned above and their interaction in the countries concerned thus influence the activity (production and use) related to 3D-printed firearms and result in the observed differences between the regions.

Although the information available on the production material was limited, useful knowledge could be gathered on how the production process was carried out by the people involved in the collected reports. As for the printing process of the seized printers, it is evident from the proportion of MEX printers observed in the cases that these machines are preferred for the manufacturing. MEX printers are widely used because of their relative simplicity, ease of use and affordability. The community around 3D-printed firearms also relies primarily on this process. Indeed, this can be observed on social networks (e.g., X formerly known as Twitter), forums (e.g., Reddit) or blogs. The general consensus is that VPP printers and their material do not meet the requirements and that MEX printers are currently the only tools that meet the requirements for 3D-printed firearm production as well as for functionality. Another disadvantage of VPP printers is that the build volume is much smaller compared to the volume of MEX printers. That VPP printers are not suitable was also proven in a scientific study in which Liberators were produced with such printers [22]. It turned out that problems already occurred during assembly stage where components started to break. Ultimately, none of these 3D-printed firearms could be discharged.

The VPP printers were models from well-known manufacturers. The identified devices are all available for less than 500 EUR. In general, the price of VPP printers has decreased in recent years, making them much more affordable. Among the MEX printers, likewise, known models could be identified. Only in the case of ten 3D printers could neither the printing process, a manufacturer nor a model be assigned. Except for a few models, the prices of the listed models are below 600 Euro. For instance, Prusa printers of the i3 series cost between 700 and 1000 EUR, Bambulab's X1 printers are available between 1000 and 1300 EUR, and Ultimaker printers can cost several thousand Euro. Among all the models, however, the Ender 3 stands out. This model can be bought in different versions and with different configurations. The starting price for the basic model is around 200 EUR, but the latest models are priced at around 550 EUR in 2023. The aforementioned prices impressively show that expensive and high-tech printers are not needed, but that users can resort to relatively cheap machines for the production of 3D-printed firearms or parts. Again, these findings are also consistent with those that can be made on the Internet. Some blogs run by well-known people in the community also refer to such “low-end” printers, most notably the Creality Ender series, but nowadays also to the more expensive Bambulab 3D printers. Furthermore, the same can often be observed in posts, images and video material shared by people on social media. These sources also lend themselves very well to being constantly informed about the material used, as this is often discussed.

Unfortunately, less precise information could be collected on the material; however, it shows a tendency. Only PLA and modified PLA (e.g., PLA+, PLA Pro) variants were observed. This material is characterised by the ease of printing and does not require any specific prerequisites of the printer, which means that this material can be used easily with most printers. More demanding materials, such as nylon or polycarbonates, require certain features (e.g., high printing temperatures, hardened nozzles) offering better physical properties of the printed parts, although these were not observed. Manuals for the manufacture of 3D-printed firearms, which are often downloaded from the Internet with the digital blueprints, also recommend using PLA type materials. This observation therefore confirms the trend seen throughout the cases. In conclusion, it can be said that no expensive or hard-to-use material is required for the production of 3D-printed firearms or parts.

The equipment can therefore be composed of rather entry-level or low-end material making the entry into 3D printing faster and easier than it would be with more complex materials. The myth that high-end material is needed to fabricate 3D-printed firearms has definitely been debunked here. This misconception has also been disproved in scientific studies on the Liberator pistol, for which these firearms were produced using both high-end and entry-level printers. In both cases, it was shown that the resulting 3D-printed firearms are functional and that, in the end, there is no need to rely on expensive equipment [21,22]. Although such high-end materials are not necessarily required, it is important to note that the use of such materials can have a positive effect and enhance the durability and performance of 3D-printed firearms. Given the rapid advancements in 3D printing technology over the last decades, these materials may become more accessible and easier to use. Therefore, their potential impact on firearm quality should not be underestimated or overlooked.

Because of the complexity in the manufacturing process, it is important that all material involved in the production is searched for and incorporated in the investigations. Examples of material that can be found on scenes of investigation have been presented in section 3.5. By analysing this material and the traces, more detailed information about the production and the people involved can be obtained. Trace materials such as tools or objects can be examined for physical traces (e.g., fingermarks, DNA) and may help in the search for the persons involved. Regarding the production, details on the procurement of the plans, the required material and the method used can be discovered. Lastly, it must also be mentioned that the manufacturing sites can be multiple and therefore spread over several physical locations. In these cases, it is important that related production sites are discovered and connected.

In general, motivations for making PMF are various, being political, economic, social, cultural or historical in nature [1]. In the cases collated during this study, the motivations were generally not detailed, but when they were, they reflected a variety of concerns. The attack in Halle, Germany and another case in the Netherlands highlight cases of right-wing extremism and suggest that political motives led to their production [5,25]. Cases in the context of drug trafficking or gang crime were also observed [26,38]. A specific case is the use of 3D-printed firearms in Myanmar, where the rebels do have more restricted access to conventional firearms and thus have to resort to alternative firearms.

In summary, the project presented here focused on a description of the current situation and use of 3D-printed firearms and firearm parts. The results thus revealed allow to get a glance into the (illegal) production and use of such objects. Besides spatial and temporal information, details on the type of 3D-printed firearms and parts as well as on their production could be obtained. On one hand, the information about the use of 3D-printed firearms and parts can be considered as valuable intelligence that can be integrated in (future) forensic case work. It can provide insight on the circumstances in which cases are described and which 3D-printed objects are involved. More globally, the integration of such intelligence can help in decision-making processes and provide important investigative leads [39]. Therefore, it seems important that this sort of overview and analysis of real cases should be continued and kept up to date, allowing for the discovery of and response to possible trends and innovations in the situation. It would also be beneficial to have a larger-scale exchange of information between different organisations working or conducting research in this field. On the other hand, the presented results have scientific value. Future research in the field of 3D-printed firearms can adapt the experimental conditions to the observations described here, allowing research outcomes to be relevant with the reality on the ground.

For future work, it is important that studies can also be conducted in a sociological and criminological context on the individuals producing such 3D-printed firearms to understand more about their profile and motivation. Combining knowledge across the different fields is considered a priority to understand the sometimes complicated interaction of different technologies and subjects related to 3D-printed firearms. Priority should also be given to provide training on 3D-printed firearms and components to law enforcement personnel in order to raise awareness of this phenomenon. This will allow such 3D-printed objects to be correctly identified and classified by personnel at different levels (e.g., patrol officers, crime scene investigator, etc.) and thus protocols can be adapted, and experts called in from the beginning if necessary.

## 5 Conclusion

The existence of 3D-printed firearms has been discussed time and again in various media since the first 3D-printed firearm, the Liberator, appeared in 2013. One cannot overlook the fact that a lot of progress has been made in this field for almost a decade. The selection ranges from relatively simple constructions to designs that come close to the performance of a conventional firearm. In the meantime, the community has also grown and become strong largely thanks to the Internet, which has facilitated the sharing of knowledge and experience. Despite these developments and the growth of this activity, relatively little is yet known about the actual use and circumstances of cases faced by law enforcement agencies. The project presented here aimed to provide a current overview of the situation law enforcement agencies are facing in regard to 3D-printed firearms, as it is reported through various, and mostly public sources.

A total of 186 cases were collected from the start of 2021 to August 2023, with a majority reported in Oceania, Europe, and North America. These regions have experienced a significant rise in recorded cases since 2021. Factors such as applicable laws, politics, and cultural influences shape the occurrence and circumstances of these cases, including the type of illicit activity and the objects involved. Notably, entry-level equipment is commonly utilised for production, proving sufficient to create functional 3D-printed firearms. Although it has been considered a seemingly marginal phenomenon so far, it is evident that cases are being reported worldwide and these 3D-printed firearms or components can be employed in various contexts. Ultimately, the data reveals that 3D-printed firearms are emerging as a viable alternative in various regions, irrespective of the severity of firearm regulations and culture, and not just as a last-resort option, even where access to conventional firearms is less restricted. This trend, highlighted by higher case numbers in North America, reflects their broad appeal and indicates a demand that cuts across diverse regulatory landscapes. With the continuous progress of the 3D printing technology and these 3D-printed firearms, there is a potential that their use will become more widespread in the future and that this issue will gain more importance and attention in police work.

Although, as the data suggests, the issue of 3D-printed firearms and their use appears to be marginal at present, we believe it is important to monitor its evolution. This project has collated and contextualised data, shedding light on this recently emerging issue and providing initial intelligence into a potentially escalating problem in the future. As mentioned above, a limitation of this project was that it relied primarily on open-source information from the Clear Web. It is likely that many cases remain undisclosed, and incorporating other languages in the search could reveal additional data. Despite these limitations, we are convinced that the presented results offer valuable initial insights that shed light on various aspects and aids in anticipating encounters with specific objects under different circumstances. It is, however, crucial to continue data collection and processing related to 3D-printed firearms and components. This ongoing effort ensures timely detection and response to changes or emerging trends. After all, new 3D-printed firearm types or the use of novel materials can significantly impact police and forensic work.

## CRediT authorship contribution statement

**Stefan Schaufelbühl:** Conceptualization, Data curation, Formal analysis, Investigation, Methodology, Project administration, Supervision, Visualization, Writing – original draft, Writing – review & editing. **Nicolas Florquin:** Conceptualization, Formal analysis, Investigation, Visualization, Writing – original draft, Writing – review & editing. **Denis Werner:** Conceptualization, Formal analysis, Investigation, Visualization, Writing – original draft, Writing – review & editing. **Olivier Delémont:** Conceptualization, Formal analysis, Investigation, Methodology, Project administration, Supervision, Visualization, Writing – original draft, Writing – review & editing.

## Declaration of generative AI and AI-assisted technologies in the writing process

During the preparation of this work the authors used ChatGPT-4 in order to improve language, grammar and readability of the text. After using this tool, the authors carefully reviewed and edited the content as needed and take full responsibility for the content of the publication.

## Declaration of competing interest

None.

## Footnotes

1 More detailed information on this subdivision can be found in section 3.4.

2 The cited figure does not necessarily represent the real situation and global distribution of cases as mostly information from publicly available sources is used. Nevertheless, this figure reveals that various countries are confronted with the challenges posed by 3D-printed firearms.

3 More information on the different types of 3D-printed firearms can be found in section 3.4.
